# Context, Timing and Individualized Care: A Realist Evaluation of Safety Planning for Individuals Living with Suicide-Related Thoughts and Behaviours, Their Families and Friends and Service Providers

**DOI:** 10.3390/jcm14124047

**Published:** 2025-06-07

**Authors:** Elisa Hollenberg, Hwayeon Danielle Shin, Nadine Reid, Vicky Stergiopoulos, Laurent Lestage, Gina Nicoll, Alyna Walji, Juveria Zaheer

**Affiliations:** 1 Centre for Addiction and Mental Health, 1001 Queen Street West, Toronto, ON M6J 1H4, Canada; elisa.hollenberg@camh.ca (E.H.); danielle.shin@camh.ca (H.D.S.); nadine.reid@camh.ca (N.R.); vicky.stergiopoulos@camh.ca (V.S.); regina.nicoll@camh.ca (G.N.); alyna.walji@camh.ca (A.W.); 2 Institute of Health Policy, Management and Evaluation, University of Toronto, 155 College Street, 4th Floor, Toronto, ON M5T 3M6, Canada; 3 Department of Psychiatry, Temerty Faculty of Medicine, University of Toronto, 250 College Street, 8th Floor, Toronto, ON M5T 1R8, Canada; 4 Département de Psychiatrie et D’addictologie, Université de Montréal, Pavillon Roger-Gaudry 2900, Boulevard Édouard-Montpetit, Montréal, QC H3T 1J4, Canada; laurent.lestage@umontreal.ca

**Keywords:** suicide prevention, realist evaluation, complex interventions

## Abstract

**Background/Objectives**: This research aimed to identify and investigate how context facilitates or hinders safety planning interventions (SPIs) intended to manage suicidal ideation (SI) and behaviour (SB) from the perspective of service users, friends, family members, service providers and other key informants. Additionally, this research aimed to identify underlying mechanisms influencing the effective and acceptable management of SI and SB across these groups. **Methods**: A realist evaluation framework (i.e., Context + Mechanism = Outcome; CMO) was used to inform the qualitative study design, which explores whether SPIs are perceived as effective (i.e., outcome) and examines the underlying mechanism(s) and specific contexts involved. A total of 28 service users, 11 family members or friends and 15 key informants, including service providers and other stakeholders, participated in semi-structured interviews. A total of 18 frontline service providers also participated in three focus groups. These interviews and focus groups were analyzed to develop a shared model capturing the mechanisms and contexts influencing effective SPI implementation. Data was collected between September 2019 and December 2021. **Results**: The model consists of three pillars: (1) understanding the importance of context, timing and relationships in safety planning, (2) identifying perceived barriers and facilitators to safety planning as described by service users, family members and service providers, (3) bridging the gap between evidence and experience in implementing safety planning interventions. **Conclusions**: While SPIs are evidence-based interventions, contextual factors and perceived barriers and facilitators can impact implementation and outcomes in mental health care settings. Understanding these factors can help to explain differences in outcomes within and across patient populations and care settings, and addressing perceived challenges can improve implementation and experiences for service users, family members and service providers.

## 1. Introduction

Suicide is a major health concern internationally, and it has been reported that 700,000 people die by suicide every year [1,2]. In Canada, the estimated economic cost per death, resulting from reduced productivity, increased health service utilization, disability and premature death, exceeds CAN 1,000,000 [3]. Experiencing suicidal thoughts or behaviours is painful and distressing and requires treatment and support; these experiences are associated with high rates of comorbidity, marginalization, premature mortality and poor access to mental health care [4]. Safety planning interventions (SPIs) are considered one of the best available brief interventions in suicide prevention and are well supported by existing evidence [5,6,7]. SPIs are a collaborative process between health care professionals and service users working together to develop a plan to manage suicidal ideation (SI) and behaviour (SB). The main elements of the safety plan as originally developed include identifying the following: (1) warning signs, (2) internal coping strategies, (3) socialization strategies for distraction and support, (4) social contacts for assistance in resolving suicidal crises, (5) professional and agency contacts to help resolve suicidal crises, (6) means restriction [8]. SPIs have been shown to decrease suicidal behaviour by up to 45% [9].

Two systematic reviews [6,7] and one meta-analysis [5] illustrate the positive effects of SPIs in reducing suicidal ideation (SI) or suicidal behaviour (SB) based on outcomes that evaluate the intervention as a whole. However, limited qualitative research explores why and how SPIs work and the perceived feasibility, effectiveness and acceptability of their component elements [6,10]. Qualitative studies have been heterogeneous, examining SPIs in different contexts (e.g., perceptions of SPI at discharge from the emergency department (ED) with brief follow-up contact phone calls [11,12,13], SPI prior to discharge from inpatient units [14,15,16], in therapeutic groups [17], using online apps [18], in specialized programs for veterans [19,20,21], in community outpatient services [22] or in peer support programs [23]). These studies have focused on a range of demographic groups including veterans on their own [13,19] or with providers and/or family members [17,20,23]; health care providers for veterans [12,16]; youth and their family members [15]; the general population presenting at the ED [11] or discharged [14] from general hospitals; youth, adults, family members and clinicians for SPI app users [18]; and staff in a program for people of refugee and asylum-seeker backgrounds [22].

There are several evidence-based suicide prevention interventions beyond SPI, including pharmacological treatments. In emergency departments, medications such as antidepressants are considered an important part of suicide prevention care due to their therapeutic effects, while taking care to limit access to potentially harmful substances [24]. If a patient diagnosed with a psychiatric illness is already taking medication and is responding well to it, the emergency department may initiate prescriptions, with plans for close follow-up care after discharge [24]. However, the evidence supporting the effectiveness of some psychiatric medications for suicide prevention remains inconclusive. For example, lithium has historically been recommended for managing depressive symptoms and reducing suicide risk, but recent studies have presented conflicting findings regarding its effectiveness [25]. While there is an ongoing debate about this, for treatment-resistant depression—when patients fail to respond to two antidepressants—an Italian expert panel reached a strong consensus on the use of lithium [26]. However, in Canada, its use has declined over the past decade [27] and also remains limited in the United States, possibly due to concerns about renal toxicity and the aggressive marketing of alternative medications [28].

While mental health diagnoses are commonly understood as risk factors for suicide, it is critical to acknowledge that suicide can also occur among individuals without a formal diagnosis. In a retrospective analysis of 174,001 suicide decedents from 37 U.S. states between 2003 and 2017, 58.2% had no known mental illness [29]. This broadens the scope of prevention efforts and challenges assumptions that tie suicide risk exclusively to diagnosable mental illness. In this context, the present paper contributes to the literature by moving beyond the question of what works in suicide prevention. Focusing on SPI, the paper asks under what circumstances interventions are effective, recognizing the importance of tailoring strategies to the varied realities of those at risk.

### Research Aim and Questions

This study aimed to better understand experiences of safety planning in health care settings across Ontario, Canada. A realist evaluation framework was used to identify and investigate how context facilitates or hinders SPIs intended to manage SI and SB from the perspective of service users, friends, families, service providers and other supports. We interviewed participants from a variety of settings to better understand the specific contextual factors related to emergency care and beyond for implementing SPIs.

Specific research questions included the following:

(1) What are the experiences of individuals (context) who have experienced SI and/or SB regarding safety planning interventions that may interact, influence, modify, facilitate or hinder the intervention and its outcome?
(2) What are the components of safety planning interventions (mechanism-resource—key elements of the SPI itself and/or its implementation) that are perceived to be helpful or unhelpful for individuals (mechanism-reasoning—the human response to SPIs) who have experienced SI and/or SB?
(3) What are the perspectives of families, friends, caregivers and service providers who have supported someone who has experienced SI and/or SB, in relation to the context, mechanisms and outcomes of safety planning interventions?

## 2. Materials and Methods

### 2.1. Realist Evaluation Approach

A realist evaluation design [30] addressed the research aims above. One major assumption of this methodology is that interventions work differently in different contexts for different people, which aligns well with suicide, SI and SB being understood as complex phenomena with psychological, biological, social and cultural underpinnings. Prevention strategies that work for one individual may not work for or work differently for another. Suicide-specific interventions, including SPIs, are assumed to be complex interventions, involving multiple interacting components, feedback mechanisms and alternative and simultaneous causal strands [31].

Realist evaluation uses the CMO (i.e., Context + Mechanism = Outcome) framework [30]. The resulting CMO configurations articulate whether SPI works or does not work (i.e., outcome) in the presence of the underlying mechanism(s) taking place in particular contexts (Table 1). This manuscript was prepared using the RAMESES II reporting standards for realist evaluations (Supplemental File S1, Table S1) [32] and the COREQ checklist for qualitative research reporting (Supplemental File S2, Table S2) [33].

### 2.2. Sampling and Recruitment

#### 2.2.1. Service Users, Friends, Family Members and Other Supports

Convenience sampling for approximately 20 service users and 10–20 friends, family members and other supports was employed by posting informational study flyers on research boards at the Centre for Addiction and Mental Health (CAMH) and at the Canadian Mental Health Association (CMHA) in Toronto, Ontario, Canada. CAMH is Canada’s largest mental health teaching hospital and home to the only standalone psychiatric ED in Ontario. CMHA is a mental health community care setting. The study team contacted service providers in these settings and notified them about the study, inviting them to refer interested individuals. In such instances, service providers provided potential participants with a copy of the study flyer and study information sheet containing the study contact information. Potential participants could independently contact the study team or, if they wished, their verbal consent would be obtained by their service provider, to later be contacted by the study team for further information and enrollment. The study was also posted on the CAMH research registry and CAMH ‘Research Connect’ webpage that hosts a database of CAMH studies, with a link to the study poster. Other recruitment strategies for this group included social media posts on ‘X’.

To be eligible to participate, service users needed to be older than 18 years, have had at least one occurrence of SI or SB in their lifetime and live in Ontario. Service users were ineligible if they could not speak English fluently, were acutely ill or hospitalized, had significant visual, auditory or cognitive impairments or lived outside of Ontario. Friends or family members, including individuals bereaved by suicide, were eligible if they were currently or had previously supported a service user who had experienced SI or SB. Participants in this subgroup were ineligible if they could not speak English fluently.

#### 2.2.2. Key Informants and Service Providers

Key informants at CAMH and CMHA were purposively identified for an approximate sample of 10 participants by the study team for interviews according to known areas of expertise and experience and then contacted by the principal investigator (PI) by e-mail with a personalized request to participate, the electronic study poster attached and a connection to the study coordinator.

To recruit an approximate sample of 20 to 30 service providers for focus groups, convenience sampling was used by posting a series of study flyers in CAMH staff rooms as permitted, such that service providers could self-refer. Further, the PI was provided with a list of physicians and allied health staff by CAMH and CMHA programs and sent a personalized request by e-mail for programs to participate; an electronic version of the study poster was included in which interested participants were directed to contact the study coordinator.

To be eligible to participate in a focus group (service providers) or interview (key informants), participants had a role as a leader, physician or allied health worker experienced in working in a health care setting providing care for people who are experiencing SI and/or SB.

Ethics approval was granted by the Research Ethics Board (REB) at CAMH in Toronto, Ontario, Canada, on 1 August 2019 (REB#041-2019).

### 2.3. Data Collection

Informed consent was obtained from all participants. Semi-structured interviews were conducted with key informants, service users, friends and family members to explore experiences with SI and/or SB or in providing or organizing care and experiences with suicide interventions such as safety planning. Interviews were conducted remotely due to COVID-19 pandemic restrictions with service users and friends or family members [by J.Z.] between November 2020 and December 2021 by videoconference or phone. Data was collected from key informants (interviews) and service providers (focus groups) [by N.R., J.Z. and E.H.] in person between September 2019 and March 2020 and remotely between February 2021 and August 2021. Only participants and researchers were present during interviews and focus groups. Participants were asked about their previous experiences with safety planning in any context in Ontario, Canada, what was most helpful, least helpful, most challenging and why, for their individual help-seeking and care, caregiving or service delivery experiences. They were subsequently shown a copy of the CAMH Emergency Department SPI template adapted from Stanley and Brown [8] (see Appendix A) and asked for their thoughts and about anything that could be added or modified to make it more helpful. The interview guide was developed and reviewed in collaboration with a team member with lived experience [G.N.].

Interviews with service users, friends and family members lasted between 30 and 90 min. At the end of each interview, participants’ demographic characteristics were captured with a demographic survey administered by the interviewer. Interviews with key informants and focus groups with service providers were between 60 and 90 min long. Demographics were captured after these sessions with an online survey. Based on these survey results, the researchers built on the convenience sampling approach to balance the number of participants by broad gender categories (men, women) as recruitment progressed. Field notes were made following interviews and focus groups. All interviews were audio-recorded, transcribed verbatim and de-identified. Transcripts were available for participant review and revision upon request, although none were asked for. Data collection was followed by regular team discussion of developing patterns. Sampling continued until there was a consensus that there were multiple examples of similar participant experiences in relation to the research questions, adequate variation in the viewpoints expressed, rich descriptions of such experiences, with no new information or topics emerging, indicating saturation in themes at the level of sampling [36,37].

To ensure the safety of participants, several strategies were used. A three-question screening tool for suicide risk was administered at the beginning and end of each interview with service user participants. In the case of an increase in risk identified by this tool, a range of resources were readily available to both the researcher and participants including but not limited to counselling, connection to crisis lines and emergency support.

### 2.4. Data Analysis

All interview data were coded, analyzed thematically [38] and then configured using the CMO framework, aligned with the realist approach using NVivo 12 software (QSR International, Burlington, MA, USA). Analysis began with the reading and re-reading of service user transcripts by the authors E.H. and J.Z. and key informant transcripts by E.H., J.Z. and N.R. During this process, open codes were generated by each author and compared to build an initial coding strategy. Six transcripts were then coded independently, any interpretation discrepancies were resolved via discussion and new codes were generated before finalizing the coding strategy. The remaining service user and key informant interviews were coded by E.H. During the coding of the remaining transcripts, any emergent codes and sub-codes were identified and discussed between E.H. and J.Z. until a consensus was reached.

A subset of three friend and family member interviews were then read and coded by E.H. and J.Z. to determine the applicability of the final service user coding strategy. It was determined that the service user coding strategy could be applied directly, with the addition of some agreed-upon emergent codes specific to the friend and family member experience. The service user NVivo 12 coding tree was duplicated and further developed with the additional codes, and then the family member and friend transcripts were coded by E.H.

During the initial and subsequent interview coding, memos were made by E.H. and J.Z. to note insights and connections across content and codes. Coded data and memos were searched for repeated patterns of meanings to re-organize codes and compile a report with broader themes, summaries and analysis specifically for the safety planning intervention. Consensus was reached regarding thematic saturation based on the presence of recurring themes with no new ones, no additional information or relationships identified [39], with an understanding that all themes were not discussed in equal proportions by all types of participants (e.g., service users, service providers, key informants or family members). Rather, triangulation was used to add the voices of other participant groups for themes that were saturated in one group when they were discussed by participants in other groups in ways that confirmed, contrasted with or complemented views for the theme that was developed for the first group.

The steps mentioned above were completed by authors, E.H., N.R. and J.Z. as indicated, who have extensive experience in qualitative research. The overall process was supervised by J.Z., a clinician scientist who has clinical and research expertise in suicide intervention and realist evaluation. For the realist evaluation level of analysis, E.H., H.D.S. and J.Z. read and re-read the thematically coded material and memos, and re-read transcripts when necessary, to organize the identified themes into CMO configurations. To do so, coded material under each initial theme was analyzed descriptively to indicate how aspects of context, mechanism-resource, mechanism-reasoning or outcomes were represented in the data. The newly described CMO coded material was then examined by the authors to understand the extent of coded material with similar or contrasting CMO factors. Alternative CMO explanations were explored, and considerations were made as to whether new themes were necessary. Revised overarching themes and sub-themes were then developed with new CMO definitions to include data with exemplary quotes representing both similar and different viewpoints. A model with three pillars was developed to further group themes conceptually. Other authors (G.N. and N.R.) reviewed and provided feedback for these themes and the model, which were finalized through group discussion. Demographic characteristics were analyzed descriptively. A reflexivity statement [40] for the authors is contained in Appendix B.

## 3. Results

In total, 28 service users, 11 friends and family members and 15 key informants participated in an interview, for a total of 54 interviews (1 interview was with 2 family members). Three service user participants completed a second interview due to having more to share than was possible during one interview, an option discussed during the consent process. Eighteen service providers participated in three focus groups. Seven people consented but did not complete the study interview.

Half of the service users identified as women (n = 14; 50%), ranging from 20 to 59 years of age, and half identified as racialized (n = 14, 50%). All but one participant reported having at least one mental health diagnosis. A total of 82% of participants reported having two or more mental health diagnoses, demonstrating a high level of comorbidity among the population. Most friends and family members identified as women (n = 7; 64%), ranging from 26 to 78 years of age, and most of them identified as white (n = 9, 82%). No friends and family members were connected in any way to the service users interviewed. All of the friends and family members reported that the individual they supported had at least one mental health diagnosis and almost half had a substance use disorder. Please see Table 2, Table 3, Table 4, Table 5 and Table 6 for detailed demographic breakdowns.

### 3.1. Themes and CMO Configurations

This section presents the qualitative results in a model with three pillars: (1) understanding the importance of context, timing and relationships in safety planning; (2) identifying perceived barriers and facilitators to safety planning as described by service users, family members and service providers; (3) bridging the gap between evidence and experience in implementing safety planning interventions. Each pillar contains content that is further described using realist evaluation CMO concepts: context, mechanism (mechanism-resource or mechanism-reasoning) and outcome. The analysis demonstrates the influence of context and mechanisms on SPI processes.

#### 3.1.1. Model Part 1: The Importance of Context, Timing and Relationships

##### Timing

Creating a safety plan when experiencing acute SI or SB may be challenging.

When individuals experiencing SI or SB first arrive at an emergency department, they are often in an acute crisis state, and thinking beyond their current difficulties can be challenging (context). The timing of introducing safety planning (mechanism-resource), as well as the state of the service user’s condition (context), matters. One service user expressed difficulty engaging in safety planning because they were overwhelmed and did not have the emotional capacity (mechanism-reasoning).

*I will say that when I had come into the ER…they had given me a piece of paper (…) and they were like, can you fill this out? And it was about suicidal thoughts, and I was like, completely overwhelmed, and it was like, what are your coping skills? Or what keeps you safe? And when you’re in a headspace where you’re not wanting to do that, you’re like, why the hell are you giving this to me?*

(SU004)

Service providers similarly noted the difficulty for individuals in crisis to create a safety plan:

*When people are in crisis, their brains don’t work well. And to be able to kind of identify those things, the person might just say, I don’t know! I don’t know! I don’t know!*

(KI1015)

Several participants specifically discussed difficulty answering a ‘reasons for living’ question if they were experiencing intense distress.

*The one that I’ve never liked is step number 2 there, remind myself of my reasons for living (…) when you’re in the middle of a crisis, it’s not always the most practical to then say to yourself, remind myself of my reasons for living. (…) If you weren’t constantly thinking back and forth about suicide or things that are crappy, then you could probably just move on. Right?*

(SU010)

To create a thoughtful and meaningful safety plan, other conditions are required. One participant suggested that safety planning would be more effective when a person is feeling better (context).

*I don’t know if I would be in the mindset to write things down when I was, like, at my worst. Maybe if you can get them to a point where they’re a little more cheerful, you know, finding a reason to be good and healthy.*

(SU013)

##### Relationships

Safety planning as a conversation, not a checklist.

Relationships are important when developing safety plans. If safety planning is part of a broader conversation (context), it can be helpful and meaningful (resource-reasoning). However, if safety planning is reduced to just another checklist (mechanism-resource) to satisfy organizational requirements, it may lose effectiveness (outcome). One service provider stated,

*It can be something that’s meaningful, powerful, relevant, and it can be something that is just a matter of ticking some boxes and making sure they have the thing.*

(KI1015)

Another noted that a mandatory formal template might risk being reduced to a checklist:

*I worry always with checklists and so on about the seductive quality. So, instead of doing the template as part of a larger conversation, getting to know the patient, we simply ask very quickly, you know, what are your warning signs, right?*

(KI1009)

One service provider indicated that creating a safety plan in longer-term therapeutic relationships (context) allows more time to explore underlying reasons and core issues leading to SI (mechanism-resource) and opens a deeper conversation about how to manage difficult feelings and situations (outcome).

*I would say anyone who we’re acutely worried about safety leaves with a safety plan of sorts, whether it’s like, one of those forms all written out, or whether it’s like, ‘here’s what number to call or who to reach out to’. Safety planning, I would say, is an active part of any session with somebody who is acutely suicidal, for sure (….) And we have the luxury of therapy appointments with longitudinal care, where we can dig into this stuff that’s causing the suicidality. I feel like spending more time there gives you way more bang for your buck, and people leave not suicidal because you’ve actually addressed the core issues that are leading to suicidal thoughts.*

(FG3, P2)

A participant described how creating a safety plan (outcome) with a professional (mechanism-resource) between crises (context) was helpful to review what worked and what did not, and to adapt the safety plan as needed, over time (outcome).

*I’ve done it several times with different professionals. I find that useful because they can suggest things to add, or maybe when they’ve seen me in a crisis state, they’re like, oh, remember we tried this and it worked? I was just so out of it that I didn’t remember we did that.*

(SU025)

Other Relationships for Developing Safety Plans.

Several respondents discussed developing their safety plan in a group therapy context (mechanism-resource) which was helpful, because the group’s input generated more ideas (outcome).

*We did it together at the trauma therapy program. Yeah, because, like, a lot of the other women in the group had ideas that would have never occurred to me on my own. Ah, I found it helpful.*

(SU001)

Other participants had created safety plans with friends (context), sharing ideas and ways to keep safe (mechanism-reasoning) and taking more time (context) than would be possible with a health care professional.

*(…) just in terms of time and patience, because health care providers, obviously, are kind of on a fixed schedule. So, they have 15 min to do safety planning with you, and maybe you need longer than that, whereas with my friends, I’ve had situations where if we need to go to the other person’s house and get slushies at 7–11 and take 45 min to even start to safety plan, that’s okay because we have time for each other.*

(SU007)

Participants also suggested safety planning templates online (mechanism-resource) to complete (outcome) independently (context) in their own time (context), with existing examples of safety plans they could draw upon for ideas (mechanism-resource).

*I think it’s nice to have ones that you can fill out on your own, like, maybe if I was at that place where I still didn’t trust providers or if I wasn’t in therapy at a certain time, it’d be great to be able to go to a website and have blank templates, or maybe even see examples.*

(SU025)

The data showed variation in the context, timing and relationships in which safety planning can occur, particularly when an individual is feeling less distressed between crises. This process can help with insight and the recall of previous solutions, generate more ideas for keeping safe and provide more time for completion.

#### 3.1.2. Model Part 2: Perceived Facilitators and Barriers to Safety Planning

##### Individual Differences in Safety Planning

Use of safety plans during an acute phase of illness or a crisis.

Individual differences may affect the implementation of safety plans. Some participants anticipated challenges implementing plans when in an acute phase of illness or a crisis, whereas others found safety plans the most helpful at exactly these times.

The barrier of experiencing an acute crisis (context) for not only creating but using (outcome) the safety plan was mentioned by various respondents. One respondent said that they did not think individuals experiencing intense suicide-related thoughts would use a safety plan when in crisis.

*Um, yeah, I think it’s important. But it’s—the only sort of complicating thing is if you are in a really severe crisis, and you’re really, really dedicated to ending your life, I think you wouldn’t necessarily follow that.*

(SU028)

Another participant felt they would not personally use a safety plan in an acute phase:

*I don’t know how other people have dealt with using these, but when I’m in crisis again, I look at that and I’m like, I don’t care. And you can make the plan, but the likelihood of somebody using it is, at least for myself, is very slim. They’re like, you can use this if you’re suicidal, and I’m like, no!*

(SU004)

Another described the difficulty of reaching out for help or emerging from their despair, meaning that even if they knew what resources were available, they might not use them.

*I found that sometimes you don’t want to get better, like you don’t want to see the positives, you don’t want to see the light, and kind of get yourself out of it, you just want to kind of mope in that feeling, not that it’s any fault of your own, but you’re kind of stuck there and you want to hurt yourself sometimes, and you are thinking about that. And you kind of don’t want the help or to reach out.*

(SU011)

A family member similarly expressed concern that an individual may not use a safety plan if they are experiencing acute distress.

*Well, if you say it and then write things down (…) if you have some contact numbers, it might do some good. But if a person is really wanting to end it—they really want to take their life, you know.*

(F006)

Alternatively, other participants described safety plans as protective when experiencing suicidal thoughts:

*I think it is helpful for me. I mean, I have a bit of a mental checklist, I’m like, okay, if I start feeling terrible again, go to the ER, like just knowing that’s a possibility.*

(SU024)

*I think it’s like maybe something to fall back on when your own thoughts are dangerous.*

(SU017)

One described their safety plan as something available to them when and if they needed it:

*I love the idea of a contingency plan, so when you’re, like, down, that sounds like a great idea. I think people, as in nature, we live in waves. So, it’s bound to happen, you know, you feel great, and then tomorrow, maybe not, because that’s how it works. You know, we can’t be happy all the time, like you wouldn’t like it if it was all darkness—you wouldn’t like it if it was light all the time, right? You gotta know darkness to distinguish the light, right? Everything comes and goes. Like a wave.*

(SU013)

Diagnosis, sense of self, control and coping.

Service providers differentiated between those who would benefit (outcome) from safety planning (mechanism-resource) and those who may not, based on their diagnosis (context) or the degree to which SI is a part of an individual’s sense of self, control and coping (context).

One service provider spoke of the great value of safety planning with patients who ‘don’t usually want to die’ but are impulsive and so are scared of their suicidal thoughts.

*(…) there’s patients who actually really don’t want to die, but deal with this impulsive suicidality that comes on, and things go really dark on them really quickly. This would be more for Borderline Personality (…) and they’re actually scared by their own suicidality, and those patients actually are the best ones for safety planning, because they really want to safety plan.*

(KI1010)

For individuals with ambivalence towards living or dying, more in-depth work is needed to understand these emotions than safety planning alone. The same service provider stated,

*There’s other people who are more ambivalent (…) they won’t commit to using a safety plan because there’s still this part of them that actually wants to die. And it’s not purely impulsive, it’s more, it’s thought out and they’re really considering it (….) and so safety planning alone is not going to be effective. You need to go deeper and really try to work at the part that wants to die and try to help them understand it in a deeper way.*

(KI1010)

For people who experience chronic suicidal thoughts, they are part of their identity (mechanism-reasoning), and safety planning needs to be approached differently:

*For her, it’s part of her identity, and part of who she is, and it’s a control issue to maintain her ownership of her suicidality. And she does not want that fixed, and she does not want that examined and she does not like that kind of planning. So, when I do safety planning with her, in quotes, it’s like what are you doing this week? Are you going to therapy next week?*

(KI1014)

Another service provider discussed challenges they have experienced in safety planning with individuals with chronic suicidal ideation:

*For a large number of folks with chronic suicidality, the response is so automatic that having this safety plan, they don’t access it. So, it’s something that they’re actually doing (…) for the clinician (…) and not for themselves, ultimately.*

(FG3, P3)

##### Family and Social Networks

Positive support from family and social networks.

The social network (context) of individuals living with suicide-related thoughts and behaviours (e.g., families and friends who are involved in providing longer-term care) is often engaged through safety planning (mechanism-resource) to provide support or implement key aspects of the safety plan (outcome).

Several respondents listed family members or friends as key contacts on their safety plans. These contacts can remind individuals with SI and SB to notice warning signs and practice coping skills, provide direct emotional support and encourage them to seek help when needed.

*(…) it’s like a plan of, like, tangible actions to take if you feel that you’re in a mental health crisis, and maybe suicidal, or liable to self-harm. So, in my case, a lot of it is around, sort of, the people who are my supports. So, when I’m in that moment, I always try to call somebody, like my sister or my mom, or my partner. And I don’t want to be alone. So, I’ll—if my roommates aren’t home, I would ask to go over to somebody’s house.*

(SU028)

Sometimes, service users made specific plans in advance, with their friends, for how they might provide support. One participant explained how a plan was shared and then implemented with their inner circle:

*I circulated it to my inner circle, my friends, so that they would know what to do, how to help me, because I think everybody’s unique and you know, when I’m in a particular zone, maybe I just need them to listen rather than go, oh, you know, it’s not that bad, you know, it could be much worse or something.*

(SU018)

A service provider similarly suggested that the safety plan be shared with family and friends to involve them in how best to respond:

*We do the wellness planning, and talk about involving loved ones in either in the planning or share the plan with them. I’ll often say, you know, this is a chance for you to talk to your family, parents, partner, how do you want them to respond.*

(KI1015)

Lack of family or social networks.

When individuals are faced with worsening suicidal thoughts/behaviours, social support (mechanism-resource) can help individuals to put their safety plan into practice (outcome) for social distraction (mechanism-resource) or direct support (mechanism-resource) during a suicidal crisis. However, when individuals lack a trusted social network (context), these mechanisms become unavailable:

*I was asked at [hospital #1] to do a safety plan… They would ask about people that I could contact when I’m feeling down or feeling at risk, but I don’t feel like I have someone that I truly trust to be in that role. So, I never found that my safety plan is sufficient.*

(SU005)

*I think it’s a useful tool, just because it reminds you of people that you have there for you, and things that help, that are immediately accessible. But I also acknowledge that at some points in my life, it was harder because I did not have people to put on there.*

(SU008)

In addition, the ‘reaching out’ aspect of a safety plan (mechanism-reasoning) might be challenging for individuals who experience difficulties with social interactions (context):

*It’s been hard for me to follow the plans that have just been like, call someone, or speak to someone, because of—oh, oh, part of the general anxiety.*

(SU017)

Lack of support for friends and family members to help implement a safety plan.

Individuals who support someone with suicide-related thoughts or behaviours to implement a safety plan (mechanism-resource) may encounter difficulties (outcome) if they do not have adequate information, resources, time, the capacity or do not feel safe enough (context) to help implement the plan.

Friends and family members find it helpful to be involved in the safety planning process, especially if they are invited to be part of the process at the beginning. However, they acknowledge that there can be challenges to implementing a safety plan. Several friends and family members expressed, for example, that they cannot be present 24/7 to ensure safety.

*I couldn’t be there 24/7 and my siblings tried to be and were probably mad at me because I wasn’t. But you can’t be there if—you can’t watch someone 24 h a day.*

(F004)

Some friends and family members reported wanting to help, but ultimately felt uncomfortable or even unsafe (context) being involved in care.

*Because once it [the situation with their family member] reached physical violence, she [a therapist] was just like, you need out right now… So, for me, it’s like I can do it, I can do it. I think that was my downfall, is because I believed that I could do it so much that I ended up causing myself more trauma. I really felt like I could do it all. Until it reached the point where I couldn’t, and then I crashed.*

(F009)

A few friends and family members provided examples of times when having access to a safety plan on its own was not enough for them to fully understand the situation (context), when the person they supported was discharged to their care. In one case, the plan on its own (mechanism-resource) was not enough to fully understand the situation (context), since the service user had not disclosed their suicide-related thoughts or behaviours. An individual may not wish to disclose such thoughts, due to stigma or shame, to protect the friend or family member in question or any other variety of reasons.

*[She] gave us the papers but she had blocked off things. What she blocked off was the beginning of it where it says, I came to [hospital] after a serious attempt on my life. She cut that off… and gave us the photocopy copy of her safety plan…. We never got the picture. We never connected the dots. And that’s where I have such regret because even with that information, this happened. I read her safety plan. And I thought [the family member] was doing really well. Even with having that safety plan in place, I was naive, and I was feeling like [the family member] was doing well. And I was so wrong. I was so wrong.*

(F008)

Another family member similarly expressed that they did not know the level of severity of the situation. She echoed a need for family members to have more complete information about the situation of the person they support.

*I think it would have been helpful for her and me, you know. That’s something you do with the patient and whomever they’re living with, their caregiver. And we could discuss it. You know, I think, going back to (…) I knew at the end she was trying to protect me, and she wasn’t telling me everything. But if there was a forum where she could be open, it was encouraged with a doctor, any kind of support person, where the three of us, or you know, talk about that together, I think that would be… because then you can talk to one another more easily.*

(F001)

Some service providers concurred that family members were not often involved in safety planning and suggested that barriers like the time required, obtaining consent and confidentiality made collaboration more difficult:

*I can’t even think, like, maybe a few examples in our day treatment service, where we’ve actually pulled family into the suicide risk assessment, right? Maybe not the assessment so much as like the safety planning. [I: I imagine one of the barriers is time?] Time and then, I think like, consent and willingness. [I: Confidentiality?] Confidentiality, yeah.*

(KI1004)

Others considered the involvement of family members an important area of program development:

*There’s also potential to grow, in terms of having more safety plan[ing] and involving family members.*

(FG1, P2)

In other program contexts, the involvement of family members was more common, and these barriers were not as apparent:

*We’re approaching it with them almost like, your safety plan is like first aid for suicidal thoughts, when these thoughts hit you go into action with doing these steps. And the families come onboard and they learn the safety plan, and I get them to put it on their phone and they print out copies and really visualizing them using it.*

(KI1010)

In adolescent care contexts, the involvement of family members was considered a key aspect of safety planning:

*We create a safety plan often with these high-risk kids, with the collaboration with their parents. (….) [In] family meetings (…) I always tell youth that safety is not a secret. So It’s not something I’m going to keep secret from your family, whether you like it or not. So suicide is always something we talk frankly about in family meetings and with parents.*

(KI1008)

In summary, although friends and family members were generally in support of safety plans, they realized that depending on their personal context, access to information, ability to provide support and the context of the individual, safety plans may not be possible to implement as intended.

#### 3.1.3. Model Part 3: Bridging the Gap Between Evidence and Experience in Implementation

##### Creating Personalized Tailored Safety Plans in a Preferred Format Helps with Use

It is helpful to create a personalized safety plan for content and format such that it is more effective for the individual (mechanism-reasoning), with it also being easy to access (context) such that they are more likely to use it (outcome).

One participant spoke of the importance of creating a safety plan with elements that are already part of their everyday life (mechanism-reasoning) such that their plan is simply a reminder (outcome) to access the supports that they would normally use (context).

*(…) I’ve made mine very much real life. Like it’s things that are in my house, my friends’ numbers are on there, like we’ve worked out, sort of, when I’ll call them for support what they might do … they’re things that I would already would naturally be doing, they’re just sort of written down on a paper. So, I see it more as a reminder than something that, like, I’m forced to do or have to do.*

(SU025)

The format could also be individualized. Some structured their safety plan as a personalized visual ‘mind-map’ or flowchart. These participants planned multiple options (mechanism-reasoning) in case some pathways did not provide the support needed (outcome).

*I tend to think of safety planning almost as a mind map, where it’s like if this happens then do this, and if this happens, then you go this way. (…) So, it’s like a flowchart … I think that it can adapt to how my circumstances might change, and it makes it easier to follow because when I’m in a lot of distress, I really need it clearly laid out, like this is my next step… I always make sure that I have multiple options so there’s never one end choice, because sometimes things don’t work…So, I try to structure it in a way where I’m never going to get to a point where, like, okay, this was my last option, because that’s gonna be unsafe for me.*

(SU007)

The location of where the plan (mechanism-resource) was accessed (context) was also important, to ensure it could be easily found (outcome) during a crisis. One individual stated that storing their safety plan in a meaningful consistent location helped with its use.

*(…) it’s a quick reference, because when I’m like that emotional, I’m not really logical anymore, it’s hard for me to remember things, but I know where my paper is. Or for me, it’s in my art books, and I know what page to flip to, and then it’s something I know and I’m familiar with, so that’s kind of comforting, like, yeah, I’ve done this before, I start here, and then I try this.*

(SU025)

Others liked an idea introduced by the interviewer of creating a safety plan on an app (mechanism-resource) where they could find it easily (outcome), although they had not accessed one in this way before.

*I think an app is really helpful and [hospital] actually had an app that I used for similar things. And like, mood tracking and tracking triggers and that was really helpful.*

(SU008)

Others preferred pen and paper (context) in relation to format:

*Maybe I’m still a little, I’m more pen and paper than most people, I think.*

(SU024)

*I’m sort of a visual person. So, if I had a plan written somewhere, and I could visualize the plan.*

(SU015)

##### Rating Systems Contribute to the Implementation of Safety Plans

Various participants discussed incorporating a self-rating system (mechanism-resource) into their safety plans, to identify ‘warning signs’ of an increase in emotional distress (context), such that they could implement steps to keep themselves safe, prior to encountering a crisis situation (outcomes). The rating system could be conducted independently or in collaboration with others. One participant stated,

*There’s different levels [in the safety plan]. So, like, up until about a 9.5 out of 10, I can help myself. And if it gets to a 9.6, that’s when I need to call on the professionals.*

(SU025)

Other participants built a rating system that was then shared with a friend, family member or health care provider whom they saw regularly. As part of the plan, agreed-upon steps could be taken if an individual’s rating reached a certain level. One participant explained how they used this rating system with a friend:

*I definitely have, my safety plan, in terms of when I know I’m getting to a point of mental distress, I feel like I have that conversation with my best friend, where I give him a barometer of what I’m feeling. So, like, 0 being like, my batteries are out, I’m zero, I’m no life, I’m going to commit suicide, and 8 being the happiest I’ve ever been on earth. So, every so often, you know, I face challenges just like everybody else, my friend will ask me, hey, where are you on your scale? And like, I’ll say straight up, like, I feel like I’m a 2 today, and then my best friend knows to jump into action.*

(SU002)

An example of the use of an informal rating system used with a trusted health professional who has been seen over the long term was discussed by another participant:

*Late last week, I definitely went through a hard time. And I called his office, he called me back, he’s like, I can just tell in your voice, and he’s like, what’s your rating? And he trusts me that that rating is very accurate. And so it helps to have that, where we have an established rating system, but I bring it up when I feel I need to (…) you just have a code of, like, no, this is serious now.*

(SU025)

Service providers also endorsed the value of incorporating rating systems into safety plans, especially for individuals with ongoing or chronic experiences of suicidal thoughts and behaviours that can occasionally become acute. The respondent calls this an ‘acute-upon-chronic’ situation and may use advance directives, as described below:

*I think it’s sometimes difficult with the chronically suicidal folks. I have a lot of patients that constantly think about ending their lives, or constantly don’t want to be alive. I differentiate the risk when I look at their behaviours and what has changed. So, some of my patients, we have a safety plan and are very clear that if you tell me a certain thing, I’m going to send you to the Emerge, regardless of what else comes after that. So, if you tell me you’re, for example, at acute risk or you’ve done a certain thing, there’s no questions asked, you’re going to the Emerge. And it may just be for a night, but that just means that in that moment, you’re not safe. So, I have agreements with some patients around how I know whether they’re safe or unsafe.*

(KI1008)

The same provider described how colours can be effective rating systems for some individuals:

*She has colours that work for her. She’s like, if I’m in the red zone, I will try to kill myself. She came in, she said I’m in the red zone, and I was like, great, well, I guess you’re going to the Emerge. (…) this is a kid who’s made eight suicide attempts.*

(KI1008)

The above steps of implementing a rating system (mechanism-resource) in a safety plan helps with determining the next steps for the kind of support needed (outcome), at a point prior to an individual being so severely distressed that they might not be able to or wish to access support (context). That is, the ‘warning signs’ are identified through comparing them to an individualized baseline score on the safety plan such that the individual can access support as needed. In these examples, the rating system is most often established between crises, when an individual and their support person(s) are able to collaboratively discuss a shared rating scale.

## 4. Discussion

Our research findings resulted in the construction of a model with three pillars of important considerations for implementing safety plans. The relevance of context and mechanisms (realist evaluation concepts) to SPI outcomes were discussed for the themes within each pillar. We will discuss the findings in relation to the current literature to understand their broader relevance. We will also compare the findings to the Stanley and Brown SPI model [8] upon which many SPIs are based and evaluated [6], to understand the differences or similarities between lived experience and the expected functioning of widely known SPI mechanisms.

First, the results revealed that while safety planning is perceived as a potentially effective intervention, context, timing and relationships are important in creating safety plans. For example, the timing of introducing safety planning during an acute crisis (context) can present challenges. Additionally, identifying aspects like ‘reasons for living’ (mechanism) at a time of emotional distress may cause individuals to feel disregarded and they may struggle to focus upon or find authentic answers.

Challenges for individuals to engage in creating a safety plan when they are having difficulty thinking at a time of acute crisis (context) were also identified in other qualitative studies [11,16,22]. Individuals in a UK study presenting at the ED with self-harm or SI expressed difficulty engaging in safety planning due to feeling overwhelmed and that they often could not remember what was discussed once they returned home [11]. Providers further observed that readiness to engage in plan creation was often challenged by difficulty for individuals in seeing alternatives to suicide at times of acute crisis [16,22]. The issue of when to complete the safety plan is referred to in the Stanley and Brown [8] model by having the SPI occur after a risk assessment that seeks to learn what led to the current crisis, builds rapport and identifies warning signs that can be included in a safety plan. Building a trusting relationship (mechanism) is an important first step to safety planning in this model. Although building a relationship may not completely moderate the difficulty of filling out a safety plan at a time of acute crisis, it may help with the process.

The value of creating a safety plan relationally, through therapeutic conversations (mechanism) rather than viewing safety plans as a checklist, was the second important factor discussed by health providers and service users in the current study and has been widely noted elsewhere. In another study, veterans suggested that the safety plan template itself does not need modification, but to improve plan development, active listening and skillful prompting by a health care provider are required [19]. In a recent qualitative systematic review and meta-synthesis of experiences of SPIs, it was found that the quality of the therapeutic interaction was more important than the resulting plan itself [10]. Adequate time and training are considered necessary to support person-centered, collaborative discussions and to prevent the SPI process from being reduced to ‘risk mitigation’ [10] or a ‘checklist’. Importantly, in relation to the existing evidence-base for SPIs, collaborative co-creation is a core principle of interventions evaluated to have positive effects [6]. The Stanley and Brown model specifies that 20 to 45 min is the expected time required for this collaborative discussion [8].

In contrast to safety planning at the time of intense SI or SB, we found that embedding the process within existing therapeutic relationships between acute phases of illness (context) is experienced positively. This approach can invite discussion of contributing factors (context) to SI and may improve relevance over more time-constrained processes with health care providers. Other qualitative studies similarly report that ongoing SPI practices (mechanism) are positively perceived due to problem-solving discussions that are helpful to update existing plans [10,11,14,16,22]. The mechanism of continuing SPI practices in outpatient settings beyond initial plan creation is included in the Stanley and Brown model [8]. Other relational mechanisms through which safety plans can be created that are positively perceived within the current study and in the emerging literature include outpatient therapy groups or peer support [17,23].

In addition to the themes of timing, context and relationships described above, this study also describes facilitators and barriers to safety plan implementation as the second pillar of its model. For this pillar, individual-level differences (context) play an important role. Some participants strongly doubted they would be able, or were not able, to implement safety plans when in crisis (context), whereas others would consult the plan at exactly that time (mechanism-reasoning). Service users from a number of other studies similarly described much more difficulty and less motivation in implementing safety planning tools or strategies when experiencing intense distress, despair, depression or when in a ‘red zone’ or crisis [10,18,19,20].

Due to the prevalence of this finding, it has been suggested that cognitive and problem-solving abilities and self-regulatory behavioural coping strategies that are key to the overall SPI mechanism may not be as accessible in times of crisis [10,18]. Consequently, relying on other SPI mechanisms such as means-restrictions or family support is emphasized in some of the literature [10]. Notably, such cognitive barriers to implementing a safety plan while in crisis are anticipated in the original SPI design by Stanley and Brown [8]. The intervention suggests that after initial plan development but before implementation, clinicians troubleshoot potential barriers, prioritizing the strategies most likely or least likely to be used. Despite this mechanism, based on this study and others, using safety plans at a time of crisis remains difficult for some individuals. In the Recommendations Section to follow, adding a safety rating system or linking warning signs to strategies in SPIs are two mechanisms suggested to enhance SPIs. Such steps may help to prevent situations from escalating to a crisis when safety plans are not as useful for some people, and also to reduce cognitive load with specific advance directives should a crisis occur.

Another facilitator or barrier to SPIs was noted by service providers in relation to individual differences (context) that affect SPI implementation based on working with persons who struggle with SI but do not want to end their lives, others who are ambivalent or those for whom SI is related to core coping mechanisms. Notably, different individual characteristics and diagnoses were identified in other qualitative studies than in the current study as facilitators or barriers to SPI implementation [11,16]. Due to the disparity in perception, more research is needed to understand the importance of individual differences for SPI implementation. Furthermore, clinical supervision is suggested to address fixed beliefs by providers about the value of SPI for individuals with certain diagnoses, fears about possible negative effects of safety planning (e.g., evoking trauma) and other factors [11,14,22]. The Stanley and Brown SPI model includes an explanation that the use of the SPI may need to be adapted depending on the population, but it does not deeply examine the impact of individual differences. Many social and environmental factors place individuals at a disproportionate risk of SI and/or SB. One example is the link between childhood maltreatment and self-injurious thoughts and behaviours [41,42]; therefore, trauma-informed approaches to SPI may be relevant for tailoring interventions for this group. The findings of the current study and others substantiate the importance of individual differences as a barrier or facilitator to the functioning of SPI mechanisms.

In the second pillar exploring barriers and facilitators to SPIs, positive support from family and social networks was another key facilitator of safety plan implementation. Friends and family were often named as contacts in safety plans, acting as a critical mechanism (e.g., recognizing and responding to warning signs with social support and distraction or reminders about coping skills and other strategies). Sharing safety plans with social networks in advance (mechanism) was also discussed as highly valued to develop shared understandings of how best to respond. The effectiveness of this mechanism was strongly echoed by participants in other qualitative studies who sought support from friends or family when warning signs arose, for social distraction, to help ensure safety, to provide assistance in contacting mental health resources and for overall plan implementation [10,19,20,21].

While social network involvement is a key mechanism of SPIs, barriers to implementing this factor due to sparse networks (context) are not discussed in the Stanley and Brown SPI model [8]. The current qualitative study and others clearly demonstrate that some individuals have very few trusted family members, friends or social supports they can include in a safety plan [10,14,16,18,19,22,23]. A suggested intervention (mechanism-resource) from the literature to address this difficulty is to support service users to build new relationships or revitalize old ones [19]; once the individual’s social context is shifted, the social support mechanism in safety planning can be more easily accessed.

At times, family members or friends felt unable to implement a plan due to a lack of information, resources, time, capacity or feeling safe enough to do so (context). This finding was echoed by recommendations by family members in another study that more information and mental health psychoeducation would help to make their involvement more useful [20]. The inclusion of friends or family members in SPI processes was variable in the current study, depending on the organizational context, but was seen as a valuable area for program improvement. Overall, friend or family member involvement in SPI processes (e.g., initial safety plan creation, at review appointments or receiving support from providers) is not part of the Stanley and Brown SPI model [8]. However, such mechanisms are widely recognized to enhance safety planning across the literature [10,15,19,20,21]. Some studies even suggest constructing separate safety plans for service users and family members [15] or modifying a service user plan to incorporate instructions for family members [20] since there are different needs and roles.

### 4.1. Recommendations

This study identified ways to improve implementation in its third pillar, ‘bridging the gap between evidence and experience in implementation’. The findings reflect that the extent to which the plan is personalized (mechanism) impacts the willingness to use the plan (outcome). Some methods to personalize include ensuring the safety plan is reviewed and up to date, formatted according to an individual’s preference (e.g., flowchart or list) (mechanism), accessible and/or portable according to individual preference (e.g., printed card, sheet of paper or electronic) (mechanism). Formatting plans flexibly with photographs, images or drawings to bridge language barriers [22], using targeted language and layouts for youth and children [15] and modified or supplemental templates for family members [15,20] are further proposed in the literature to personalize plans and optimize use.

Another key recommendation for safety planning is a rating system (mechanism) established in comparison to a personalized ‘baseline’ within a safety plan to identify ‘warning signs’ of an increase in emotional distress such that steps can be taken prior to encountering a crisis situation. Such rating systems can be used independently or with family members, friends or professionals. A similar innovation was suggested in another study with adolescents engaged to provide input for an SPI design for their demographic [15]. Like in the current study, rating scales were suggested for the rapid communication of internal states to chosen support individuals using numbers to track moods which could result in pre-determined types of support or reminders of coping skills (strategies) to prevent escalation [15].

Notably, the idea of linking ‘warning signs’ to strategies more generally (without the added mechanism of rating scales) was also suggested in the current study by participants who structured safety plans as a flowchart with ‘if this happens/then do this’ scenarios. Similar suggestions are made in other qualitative studies to improve safety plans by pairing anticipated scenarios with actions that could be used in a crisis [21] or linking warning signs and strategies in a safety plan app to provide reminders during a suicidal crisis [19,43]. Service providers also discussed the importance of making such linkages [16]. Rating scales and linking warning signs and strategies are not included in the Stanley and Brown SPI [8] and represent two innovations to prevent situations from escalating to crises and, if they do, to reduce cognitive load at that point when safety plans have been found more difficult to implement for some individuals.

A summary of the above recommendations that emerged directly from the findings in pillar three, and additional suggestions woven throughout the discussion based on comparison of the findings from other pillars with the literature, are summarized in Appendix C. Below is a table derived from this appendix with an actionable summary for clinicians and administrators (Table 7).

### 4.2. Future Directions for Research

As noted earlier, more research is needed to understand how individual differences (context) affect safety planning and what corresponding adaptations to SPIs may be needed. The CAMH Suicide Prevention Cohort Study (CAMH-SPCS), which was work that grew out of the current study, will recruit 500 individuals with SI and SB who present to the CAMH ED to better understand their trajectories and needs. A qualitative component will interview a subset across different age, gender, ethnicity and diagnosis categories and will interview one friend or family member per participant separately to understand their perspectives of evidence-based interventions, including safety planning. This study will add to the existing understanding of how safety planning can apply to different types of individuals.

Furthermore, evidence-based interventions for suicide prevention, such as SPIs, are not consistently implemented or provided to all patients who might need them [44]. More efforts are needed to implement SPIs in the right context and with the right mechanisms for each person to ensure the anticipated outcomes of SPIs can be realized. To contribute to this work, a current implementation research study will further explore barriers and facilitators in delivering SPIs from emergency department clinicians’ perspectives for alternative (app) and paper-based modalities and collaboratively design strategies to address these barriers [45]. Such efforts will provide further valuable insights into how SPIs can be optimized, adapted and effectively implemented while maintaining their intended outcomes.

### 4.3. Strengths and Limitations

The use of the realist evaluation design is a strength of this study since it examines contextual factors and mechanisms that facilitate or hinder the outcomes of safety planning interventions. This methodology has not yet been applied to SPIs. The study compares its findings to prior qualitative studies, contributing to credibility and transferability, two aspects of trustworthiness in qualitative research [46]. A further strength is the triangulation of perspectives about SPIs from a relatively large and heterogeneous sample of service user participants with those of friends, family members and service users, which further contributes to credibility and transferability. There was rigorous coding and analysis of transcribed interviews, reflexive practice through memo writing and peer review of coding and manuscript writing, which contributes to dependability and confirmability [46].

A key limitation is that although many participants shared views on safety planning, the specific type of safety planning model experienced was not explored in the primary study to know whether all of the steps were completed (e.g., Stanley and Brown SPI [8]). Thus, the safety planning interventions experienced across participants may have differed. In addition, the research goals did not include examining implementation differences across different care contexts such as emergency care, inpatient units or community follow-up. Regardless, the findings reflect very similar experiences for participants in this study as in other qualitative studies with known intervention types and care settings, which contributes to credibility and transferability. Future directions for research, moreover, include examining implementation issues in the emergency department, specifically.

A further limitation is that the study did not purposively or theoretically sample for language spoken, geographical or socio-demographic diversity and only recruited English-speaking individuals. Furthermore, while the demographic characteristics indicated diversity for gender identity, race and socio-economic factors, the findings were not analyzed to explore whether certain groups may have unique needs and preferences. While the sampling and analysis approach may limit transferability [46], this limitation was moderated by comparisons in the discussion with prior qualitative research across various demographic groups in various geographic regions that demonstrated similarities across multiple findings.

## 5. Conclusions

In this paper, qualitative interviews were used to better understand the lived experiences of individuals who have experienced SI and/or SB with SPIs. Friends, family members and health care providers were also interviewed about their views. A realist evaluation analysis distilled which contexts and mechanisms influenced SPI implementation. A resulting model was developed with three pillars: (1) understanding the importance of context, timing and relationships in safety planning; (2) understanding perceived barriers and facilitators to safety planning; (3) bridging the gap between evidence and experience in implementation. Taken together, and compared to the current literature and the Stanley and Brown SPI [8], the results demonstrate the value of certain SPI mechanisms and draw attention to others for which taking context into consideration, or adding interventions, would enhance SPI outcomes.

## Figures and Tables

**Table 1:** Realist CMO definition of terms.

Domain	Definition
Context (C)	Contexts are all factors that are not part of the intervention itself, and features of conditions in which interventions are introduced that are relevant to mechanisms’ operation [30]. Contexts interact, influence, modify, facilitate or hinder the intervention and its outcome (e.g., effectiveness) [30].
Mechanism (M)	Mechanisms are the combination of the intended and unintended resources offered by an intervention, as well as the reactions and/or responses (e.g., cognitive, emotional, motivational reasoning, physical) to those resources that make an intervention work [34]. Mechanisms can be further classified as (1) the resources provided by the program (M-resource) or (2) the human response to receiving those resources (M-reasoning) [34].
Outcome (O)	Outcomes are the results of an intervention with multiple underlying mechanisms, which can lead to different effects on individuals in various situations, resulting in the possibility of multiple outcomes [30].
	“CMO configuring is a heuristic used to generate causative explanations about outcomes in the observed data. A CMO configuration may be about the whole [intervention] or only certain aspects” [35] (p. 3).

**Table 2:** Service user demographic characteristics.

Characteristics	*n* (%)
***n***	28 (100)
**Average Age (years, range)**	32.9 (20–59)
**Gender**	
Woman ^1^	14 (50)
Man ^1^	10 (36)
Non-binary	4 (14)
**Race**	
Racialized ^2^	14 (50)
White	14 (50)
**Marital Status**	
Married/partnered	7 (25)
Single/divorced	20 (71)
**Education**	
Completed college/university	19 (68)
Less than college/university	9 (32)
**Employment**	
Employed	
Full-time	8 (29)
Part-time	6 (21)
Unemployed	13 (46)
**Source of Income ^3^**	
Average number of income sources (range)	1.2 (0–3)
Employment	13 (46)
Social support ^4^	12 (43)
Family support/savings	8 (29)
Student loan	4 (14)
**Living with**	
Alone	12 (43)
Family	7 (29)
Spouse/partner	4 (14)
Friend/roommate	3 (11)
No answer	2 (7)

^1^ Includes cis and trans women/men. ^2^ Includes East Asian, South Asian, Black-Caribbean, Black-North American, Latin American, Middle Eastern and two or more racial/ethnic groups. ^3^ Categories are not mutually exclusive. ^4^ Includes Ontario Disability Support Program (ODSP), Employment Insurance (EI), Canadian Emergency Response Benefit (CERB) and long-term disability.

**Table 3:** Mental health characteristics of service users.

Characteristics	*n* (%)
***n***	28 (100)
**Previous Emergency Department (ED) Visit**	
Yes	19 (70)
**Previous Hospitalization**	
Yes	15 (54)
Average number of hospitalizations (range)	3.1 (2–6)
**Current Diagnoses**	
Average number of diagnoses (range)	2.9 (0–6)
Depression and related ^1^	23 (82)
Anxiety disorders ^2^ and Obsessive-compulsive disorder (OCD)	17 (61)
Post-traumatic stress disorder (PTSD) and related ^3^	15 (54)
Borderline Personality Disorder	10 (36)
Bipolar Disorder	5 (18)
Substance Use Disorder	3 (11)
Attention-deficit/hyperactivity disorder (ADHD)	3 (11)
Other ^4^	4 (14)
**Comorbidity**	
Yes	23 (82)
**Treatment team**	
Primary care provider	16 (62)
Psychiatrist	11 (42)
Specialist physician	3 (12)
**Allied Health**	
Therapist	11 (44)
Social worker	3 (12)
Case worker	3 (12)
Allied health linked with psychiatry ^5^	4 (15)
Peer support	2 (8)

^1^ Includes depression, chronic depression, Major Depressive Disorder, Major Depressive Episode and Adjustment Disorder. ^2^ Includes Generalized Anxiety Disorder, Social Anxiety Disorder and Panic Disorder. ^3^ Includes PTSD, Complex post-traumatic stress disorder (CPTSD), Dissociation Disorder and Dissociative Identity Disorder. ^4^ Includes Schizophrenia, Autism Spectrum Disorder and Unspecified Eating Disorder. ^5^ Includes aftercare, online programs and care at psychiatric hospitals.

**Table 4:** Friend and family demographic characteristics.

Characteristics	*n* (%)
***n***	11 (100)
**Average Age (years, range)**	50.6 (26–78)
**Gender**	
Woman ^1^	7 (64)
Man	2 (18)
Not listed	10 (1)
**Race**	
Racialized ^2^	2 (18)
White	9 (82)
**Marital Status**	
Married/partnered	7 (64)
Single/divorced	4 (36)
**Has Children**	
Yes	6 (55)
Average number ofchildren (range)	2.7 (2–3)
**Education**	
Completed college/university	7 (36)
Less than college/university	4 (36)
**Employment**	
Employed	
Full-time	27 (3)
Part-time	2 (18)
Self-employed	2 (18)
Unemployed	2 (18)
Retired	2 (18)
**Source of Income ^3^**	
Average number of income sources (range)	1.2 (0–2)
Employment	6 (55)
Social support ^4^	2 (18)
Family support/savings	6 (55)
**Living with**	
Alone	2 (18)
Spouse/family	8 (73)
Friend/roommate	1 (9)

^1^ Includes cis and trans women/men. ^2^ Includes Black-African and two or more racial or ethnic groups. ^3^ Categories are not mutually exclusive. ^4^ Categories are not mutually exclusive.

**Table 5:** Description of friend and family mental health characteristics.

Characteristics	*n* (%)
***n***	11 (100)
**Previous Hospitalization**	
Yes	11 (100)
Average number of hospitalizations (range)	5.3 (1–20)
**Current Diagnoses**	
Average number of diagnoses (range)	1.9 (1–4)
Depression	5 (45)
Anxiety	1 (9)
PTSD	1 (9)
Borderline Personality Disorder	3 (27)
Substance Use Disorder	6 (55)
Other ^1^	4 (36)

^1^ Includes Somatic Symptom Disorder, an eating disorder, a reading disorder and psychosis.

**Table 6:** Key informant and service provider demographic characteristics.

Characteristics	*n* (%)
***n***	33 (100)
**Gender**	
Woman	25 (76)
Man	8 (24)
**Role ^1^**	
Frontline clinician ^2^	18 (55)
Physician	9 (27)
Administration/leadership	11 (33)
**Ethnicity**	
White	19 (58)
Racialized	13 (39)
Prefer not to answer	1 (3)

^1^ Categories are not mutually exclusive. ^2^ Includes nurses, social workers, occupational therapists, case managers and program managers.

**Table 7:** Recommendations for clinicians and administrators with regard to SPIs.

SPI Mechanisms	Recommendation
Introducing Safety Plans (Timing)	Complete risk assessment prior to safety planning to hear what led to the current crisis, to build rapport and to identify warning signs that can be included in a safety plan (first step in the Stanley and Brown [8] SPI). Consider the importance of timing and context when co-creating safety plans. Possibly delay, or complete risk assessment as above and establish a trusting relationship first, if in acute crisis.
Safety Plan Creation	Co-create a safety plan relationally and collaboratively rather than completing it as a ‘checklist’ to fulfill instrumental or ‘risk mitigation’ goals required by the organization. Time for safety plan completion (20–45 min recommended in the Stanley and Brown [8] SPI). SPI training in all aspects of the Stanley and Brown SPI model [8]. Active listening and skillful prompting by clinician; attend to quality of the therapeutic relationship. During safety plan creation, before finalizing the plan, troubleshoot barriers to implementation during a crisis by prioritizing the strategies most likely and least likely to be used during this time (mechanism in the Stanley and Brown [8] SPI).
Ongoing Safety Planning	Continue SPI practices within an ongoing professional therapeutic relationship in the outpatient context (recommended by the Stanley and Brown [8] SPI). Review what has worked and not worked during times of crisis; discuss things to add based on reflection (e.g., new warning signs, strategies, contacts); update plan accordingly.
Troubleshooting Implementation Issues Some service users doubt they will use safety plans in a time of crisis or have experience in not using them at this time.	During safety plan creation, before finalizing the plan, troubleshoot barriers to implementation during a crisis by prioritizing the strategies most likely and least likely to be used during a crisis situation (mechanism in the Stanley and Brown [8] SPI). Ensure means-restrictions and chosen family and/or friend supports are in place as a result of prior safety planning to accommodate inability to use safety plans during crisis situations. Add safety scales with linked strategies and/or link warning signs and strategies within SPIs. Update as needed through ongoing SPI practices. Share rating scales with chosen family members and/or friends, to ensure shared language to communicate distress and pre-determined strategies when crisis situations occur. These approaches may reduce cognitive load during a crisis and facilitate implementation of safety plan strategies that may not be normally possible due to difficulties with problem-solving and behavioural self-regulation at this time.
Adapting SPIs for Individual Differences Individual differences related to diagnosis, sense of self, control and coping may either facilitate or prevent engagement with, or use of, safety plans.	Maintain awareness of variation in responses to SPIs. Introduce safety planning to individuals who may find it helpful. Adapt SPIs to focus on the short-term or introduce other adaptations for individuals who may see SI or SB as part of their identity, sense of control or coping. As appropriate for individuals with longstanding SI and/or SB, facilitate therapeutic processes to explore root causes. Trauma-informed approaches to care and clinical supervision.
Family and Social Network Involvement	List the chosen social supports on the safety plan for social distraction or to contact during a suicidal crisis. Share the safety plan with chosen members of the social network. When trusted family members or social networks are lacking, support service users to build new relationships or revitalize old ones [19]; once the individual’s social context is shifted, the social support mechanisms in safety planning can be more easily accessed. To increase safety and effectiveness, involve chosen family members and/or friends in SPI processes with adequate information about the service user’s situation and warning signs, offer mental health psychoeducation sessions, construct separate safety plans tailored to support person needs and/or add professional contacts for family and friend support.
Formatting SPIs	The extent to which the plan is personalized and accessible impacts the willingness and ability to use the plan. Ensure the safety plan is up to date, including content formatted according to an individual’s preference (e.g., flowchart or list, language, visual cues), accessible and/or portable according to individual preference (e.g., printed wallet card, sheet of paper or electronic).

## Data Availability

The data presented in this study are available on request from the corresponding author. The data are not publicly available due to privacy and ethical restrictions.

## Supplementary Materials

The following supporting information can be downloaded at https://www.mdpi.com/article/10.3390/jcm14124047/s1, Supplemental File S1, Table S1: RAMESES II reporting standards for realist evaluations: checklist; Supplemental File S2, Table S2: Consolidated criteria for reporting qualitative studies (COREQ): 32-item checklist.

## Abbreviations

The following abbreviations are used in this manuscript:

ADHD	Attention-deficity/hyperactivity disorder
CAD	Canadian Dollar
CAMH	Centre for Addiction and Mental Health
CERB	Canadian Emergency Response Benefit
CMHA	Canadian Mental Health Association
CMO	Context + Mechanism = Outcome
CPSTD	Complex post-traumatic stress disorder
EI	Employment Insurance
ED	Emergency Department
OCD	Obsessive-compulsive disorder
ODSP	Ontario Disability Support Program
PTSD	Post-traumatic stress disorder
SI	Suicidal Ideation
SB	Suicidal Behaviours
SPIs	Safety Planning Interventions

## Appendix A

### Centre for Addiction and Mental Health (CAMH) Safety Plan Template

The following safety plan template is adapted from Stanley and Brown’s 2012 paper entitled ‘Safety Planning Intervention: A Brief Intervention to Mitigate Risk for Suicide’ [8] and the CAMH Suicide Prevention and Assessment Handbook (2011).

**Table A1:** Centre for Addiction and Mental Health (CAMH) safety plan template.

Safety Plan
**Step 1: Warning signs that I may not be safe**
1. 2. 3.
**Step 2: Remind myself of my reasons for living**
1. 2. 3.
**Step 3: Coping strategies that I use to distract myself or feel better**
1. 2. 3.
**Step 4: Social situations and people that can help distract me**
1. 2. 3.
**Step 5: People who I can ask for help**
1. 2. 3.
**Step 6: Professionals or agencies I can contact during a crisis**
1. 2. 3.
**Step 6: Professionals or agencies I can contact during a crisis**
1. 2. 3. 4. 5.
**Step 7: Making my environment safe**
1. 2. 3.

## Appendix B

### Author Reflexivity Statement

The qualitative and realist evaluation methodologies used in this study both adopt an interpretive epistemological stance, proposing that human knowledge is socially constructed. The realist methodology additionally adopts an ontological position that there is a separate ‘reality’ apart from human observation, but that this ‘reality’ can be perceived only partially, due to how it is filtered through language, culture and other factors [40]. Due to these philosophical perspectives, both research methodologies suggest that the positioning of the researchers be made transparent, in what is known as ‘reflexivity’. Reflexivity asks the researchers to examine and then share how the research context (e.g., culture, health care system, local policies or service frameworks) and the researchers’ backgrounds and perspectives may influence study design, implementation, analysis, writing and other research processes.

Due to these principles, it is important to acknowledge that a number of the research team members have worked as psychiatrists (L.L., V.S., J.Z.) or in research roles (H.D.S.) in CAMH’s Gerald Sheff and Shanitha Kachan Emergency Department (ED), Canada’s largest mental health teaching hospital and only standalone psychiatric ED in Canada. This ED receives over 15,000 visits annually, and approximately 1/3 of these visits have identified suicidal ideation (SI) or suicide-related behaviours (SB) at triage. In the CAMH ED, there is a standard SPI template adapted from Stanley and Brown [8]. Upon discharge from the ED, individuals leave with a paper-based, personalized safety plan. Several authors, therefore, were familiar with how SPIs were implemented and experienced in this context prior to the study.

One objective of the study was to understand the lived experiences of SPIs from various perspectives (service users, friends, family and service providers) and what components were helpful or not helpful in the ED context but also in other Ontario, Canada, health care and community contexts, as reflected by its broad recruitment strategy. Therefore, perceptions of the SPI process at the CAMH ED needed to be considered by some authors when collecting or analyzing the current study data. In addition to acknowledging prior experience with SPIs, the researchers’ broader backgrounds are important to acknowledge for their possible influence. Overall, the researchers brought clinical practice experience, lived experience and research experience to this study. They all have longstanding relationships within the health context in Ontario. The researchers disclosed their roles and positionality in the informed consent document and at the beginning of interviews or focus groups.

E.H. (MSW, she/her) is a research coordinator with qualitative research expertise in the area of SI and SB, a registered social worker, and has previously worked as a community mental health provider for the friends and family members of people with SI and SB, as well as being a family member herself. H.D.S. (RN, PhD, she/her) is a registered nurse with experience in emergency and mental health nursing with extensive experience in qualitative research, collaborative research and implementation research in the mental health context including suicide prevention. N.R. (PhD, she/her) is a researcher and evaluator with expertise in realist evaluation and qualitative research methods centred in equity and engagement. V.S. (MD, she/her) is a psychiatrist and health services researcher who often works with patients who have experienced SI and SB. L.L. (MD, he/him) is an emergency department psychiatrist who often works with patients who have experienced SI and SB. G.N. (she/her) is a research assistant with lived experience of mental illness and suicide and has expertise in research and peer support. A.W. (MSW, she/her) is a research analyst with qualitative research experience in mental health and addictions and a registered social worker who frequently works with people who experience SI and SB. J.Z. (MD, she/her) is an emergency department psychiatrist and researcher with qualitative research expertise. She often works with patients who have experienced SI and SB.

During the study, different views and perspectives were acknowledged and shared among research team members. This process resulted in increased reflexivity and conversations about how best to interpret the data. Consequently, one of the goals of reflexivity to broaden the conversation and to include alternate perspectives was accomplished [40].

## Appendix C

### Summary of Findings and Recommendations

**Table A2:** Summary of findings and recommendations.

Theme	Context	Mechanism	Outcome	Recommendation
**Pillar 1: Importance of Timing, Context and Relationships**				
**Timing**				
Creating a safety plan or identifying reasons for living when experiencing acute SI or SB may be challenging.	Acute crisis.	Introducing safety planning. Creating a safety plan. Discussing reasons for living.	Overwhelmed. Feeling disregarded or unheard. Struggles to focus upon or find authentic answers to safety planning questions.	Complete risk assessment prior to safety planning to hear about what led to the current crisis, to build rapport, and to identify warning signs that can be included in a safety plan (first step in the Stanley and Brown SPI). Build a trusting relationship as a first step to co-creating a safety plan. Consider the importance of timing of the intervention and context of the service user, when co-creating safety plans.
**Relationships**				
Co-create a safety plan relationally and collaboratively rather than completing it as a ‘checklist’ to fulfill instrumental or ‘risk mitigation’ goals required by the organization.	Time for safety plan completion (20–45 min recommended in the Stanley and Brown SPI). Availability of a clinician with SPI training in all aspects of the Stanley and Brown SPI model (including aspects of the SPI that are not included in the safety planning template alone, but can be found in the original intervention protocol).	Clinician and service user co-create the safety plan collaboratively. Active listening by clinician. Skillful prompting by clinician. Attend to quality of the therapeutic relationship (which is considered more important than the resulting safety plan by service users). During safety plan creation, before finalizing the plan, troubleshoot barriers to implementation during a crisis by prioritizing the strategies most likely and least likely to be used during this time (mechanism in the Stanley and Brown SPI).	Safety plan that is personalized and reflects authentic input from the service user that the individual can take with them.	See content in the adjacent context and mechanism columns for this theme.
Embed safety plan within existing therapeutic relationships.	Access to therapeutic relationship in the outpatient context. Skilled clinicians who can discuss the underlying causes of SI or SB.	Continue SPI practices within an ongoing professional therapeutic relationship in the outpatient context (recommended by the Stanley and Brown SPI when possible). Review what has worked and not worked during times of crisis; discuss things to add based on reflection (e.g., new warning signs, strategies, contacts); update plan accordingly. Engage in therapeutic discussions about the underlying causes of SI or SB.	Revised and updated safety plan. Barriers to implementation may be discussed through review. The underlying causes of SI or SB may be discussed in some contexts to help with reflection and healing.	See content in the adjacent context and mechanism columns for this theme.
Other relationships for creating safety plans.	Therapeutic group settings with a focus on SPI. Access to supportive peers who are knowledgeable about, and able to assist with safety plan creation. Access to online templates or apps for safety planning; trusted professional support may not be available.	Therapeutic groups may provide education and structure a process for safety planning with peers and clinician-facilitators. Peers may engage in informal collaborative safety planning outside of the clinical context. Service users may access online SPI tools independently, outside of the clinical context.	Safety plans are created through group processes, resulting in peer-support and generating more ideas. Safety plans are created with peer-support and possibly more time than is available in clinical contexts. Safety plans are created independently, which may be useful in ties when trusted professional support is not available.	Explore alternative relational mechanisms to create safety plans, such as therapeutic groups; peer support; and online safety planning resources that can be used independently at times when trusted professional support is not available.
**Pillar 2: Perceived Facilitators and Barriers to Safety Planning**				
**Individual Differences in Safety Planning**				
***Use of safety plans during an acute phase of illness or a crisis***				
Some service users doubt they will use safety plans in a time of crisis or have experience in not using them at this time.	Acute crisis. ‘Red zone.’ Intense depression. Lack of motivation. Despair.	Cognitive and problem-solving abilities and self-regulatory strategies to implement safety plan mechanisms are not accessible to the service user.	Safety plan not used.	During safety plan creation, before finalizing the plan, troubleshoot barriers to implementation during a crisis during by prioritizing strategies most likely and least likely to be used during a crisis situation (mechanism in the Stanley and Brown SPI). Ensure means-restrictions and family support are in place as a result of prior safety planning to accommodate inability to use safety plans during crisis situations. Add safety scales with linked strategies and/or link warning signs and strategies within SPIs. Share rating scales with support networks (including available friends, family and clinicians) to ensure there is a shared language for service users to communicate distress and pre-determined strategies to enact when crisis situations occur.
***Diagnosis, sense of self, control and coping***				
Individual differences related to diagnosis, sense of self, control and coping may either facilitate or prevent engagement with, or use of safety plans.	Individuals who struggle with SI but do not want to end their lives may find SPI helpful. Individuals who are ambivalent or for whom SI is related to identity or core coping mechanisms may not engage in SPI or find SPI helpful. Access to outpatient context for SPI practice. Skilled clinicians able to facilitate therapeutic processes to explore the root causes of SI or SB.	Introduce safety planning to individuals who may find it helpful. Adapt SPI to focus on the short-term or introduce other adaptations for individuals who may see SI or SB as part of their identity, sense of control or coping. Work with individuals with chronic SI to explore the root causes of SI or SB.	Adapted safety plan interventions to needs of specific individuals. Increased awareness and understanding of self and root causes of SI and SB for individuals who engage in therapy.	Develop or maintain awareness of variation in responses to SPIs depending on the context of the individual. Clinical supervision to help with clinician ‘fixed’ beliefs about the ability of individuals with particular diagnoses to engage in safety planning and to provide support around fears about possible negative effects of safety planning (e.g., evoking trauma).
**Family and social networks**				
***Positive support from family and social networks***				
Positive support from family and social networks is a key facilitator to safety plan implementation.	Positive relationships with a number of friends, family members and social networks that can be listed for social distraction or SI or SB crisis support on a safety plan.	List social supports on the safety plan for social distraction or to contact during a suicidal crisis, as appropriate. Share safety plan with members of the social network.	Service users will contact the friends, family members or members of their social network listed on their safety plan for support when needed. Members of the social network may recognize and respond to warming signs, provide social distraction or support during a suicidal crisis.	See content in the adjacent mechanism column for this theme.
***Lack of family or social networks***				
Individuals with sparse social networks cannot draw upon key SPI mechanisms involving social distraction or direct support for SI or SB.	Some individuals do not have any trusted friends, family members, professionals or social networks they can draw on for social distraction or support when experiencing SI or SB.	None.	Without additional interventions (see adjacent recommendation), service users will not be able to add members of their social network to their safety plan for support. Service users may see their safety plans as deficient due to their inability to use this mechanism.	Support service users to build new relationships or consider old ones; once the individual’s social context is shifted, the social support mechanisms in safety planning can be more easily accessed.
***Lack of support for friends or family members to help implement safety plan***				
At times, family members or friends felt unable to implement a plan due to lack of information, resources, time, capacity or feeling safe enough to do so (context).	Friends and family members lack information, resources, time or capacity to fulfill their roles in the SPI.	None.	Service users will not be able to fully benefit from the support of family members. Mechanisms involving friends, family members and social networks will not be available or will be deficient in some way.	Involve family members in SPI processes with adequate information about the service user’s situation and warning signs, offer mental health psychoeducation sessions, construct separate safety plans tailored to support person needs or add support person information to the service user’s safety plan.
**Pillar 3: Bridging the gap between evidence and experience in implementation**				
**Creating personalized, tailored safety plans in a preferred format helps with their use**				
The extent to which the plan is personalized and accessible impacts willingness and ability to use the plan.	Access to a collaborative co-creation process wherein a safety plan is constructed by a service user and clinician working together. Access to ongoing outpatient SPI support from a skilled clinician for ongoing safety plan review.	Through the original co-creation process and in follow-up outpatient SPI practices, ensure the safety plan is up to date, formatted according to an individual’s preference (e.g., flowchart or list) accessible and/or portable according to individual preference (e.g., printed card, sheet of paper or electronic). Ensure the safety plan is located in an accessible format or location.	Individuals will be more likely to use and implement their safety plan.	Formatting plans flexibly with photographs, images or drawings to bridge language barriers, using targeted language and layouts for youth and children and modified or supplemental templates for family members are further proposed in the literature to personalize plans and optimize use.
**Rating systems contribute to the implementation of safety plans**				
Add a rating system established in comparison to a personalized ‘baseline’ to an SPI to identify ‘warning signs’ of an increase in emotional distress such that steps can be taken prior to encountering a crisis situation. and/or: Link warning signs to strategies in safety plans.	Access to clinical support or templates to create a safety rating scale with linked strategies (and/or to link warning signs and strategies). Access to positive support from friends, family members, professionals or members of social support network who can be contacted for social distraction or support around SI and SB.	Construct a safety rating scale with linked strategies (and/or warning signs with linked strategies) with a clinician or using a template. Share completed rating scales with linked strategies (and/or SPIs with warning signs and linked strategies) to chosen friends, family members, professionals or members of social support network. Update safety rating scale and linked strategies (and/or warning signs and linked strategies) as needed through ongoing outpatient SPI practices. Service users will communicate emotional states to chosen support persons using ratings (and/or will directly discuss ‘warning signs’), as needed, or will reflect upon ratings (and/or ‘warning signs’) and linked strategies for self-regulation.	Use of the safety rating scale and linked strategies (and/or ‘warning signs with linked strategies) within the SPI may prevent escalation to crisis situations. The safety rating scale (and/or an SPI with linked warning signs and strategies) may improve communication to support persons about emotional states and result in reminders about, or implementation of, strategies associated with each rating (and/or warning sign). Pre-determined strategies related to certain ratings (and/or linked to warning sign) may result in preventing SB, with or without additional social network support. Increased awareness of ratings and/or warning signs may increase the ability to use suicide coping skills. These techniques may reduce cognitive load during a crisis and facilitate implementation of safety plan strategies that may not normally be possible due to difficulties with problem-solving and behavioural self-regulation at this time.	Create templates or draw upon existing ones to add safety rating scales with linked strategies (and/or warning signs with linked strategies) to SPIs. See content in the adjacent context and mechanism columns for additional recommendations for this theme.
