# Supplementary material for: Context, Timing and Individualized Care: A Realist Evaluation of Safety Planning for Individuals Living with Suicide-Related Thoughts and Behaviours, Their Families and Friends and Service Providers
Source: J Clin Med. 2025 Jun 7;14(12):4047. doi: 10.3390/jcm14124047 (PMC12194053; doi:10.3390/jcm14124047)
Supplement: Supplementary file 1 [file jcm-14-04047-s001.zip › jcm-3482211-supplementary.pdf]

Journal of
Clinical Medicine

MDPI

Article

Context, timing, and individualized care: A realist evaluation of safety planning for individuals living with suicide-related thoughts and behaviours, their families and friends and service providers

Elisa Hollenberg [1,†], Hwayeon Danielle Shin [1,2,†], Nadine Reid [1,2], Vicky Stergiopoulos [1,2,3], Laurent Lestage [4], Gina Nicoll [1], Alyna Walji [1] and Juveria Zaheer [1,2,3,*]

[1] Centre for Addiction and Mental Health, 1001 Queen Street West, Toronto, ON M6J 1H4; elisa.hollenberg@camh.ca (E.H.); danielle.shin@camh.ca (H.D.S.); nadine.reid@camh.ca (N.R.); vicky.stergiopoulos@camh.ca (V.S.); regina.nicoll@camh.ca (G.N.); alyna.walji@camh.ca (A.W.)
[2] Institute of Health Policy, Management and Evaluation, University of Toronto, 155 College Street, 4th Floor, Toronto, ON M5T 3M6, Canada
[3] Department of Psychiatry, Temerty Faculty of Medicine, University of Toronto, 250 College Street, 8th Floor, Toronto, ON M5T 1R8, Canada
[4] Département de psychiatrie et d'addictologie, Université de Montréal, Pavillon Roger-Gaudry 2900, Boulevard Édouard-Montpetit, Montréal, QC H3T 1J4, Canada; laurent.lestage@umontreal.ca (L.L.)

* Correspondence: juveria.zaheer@camh.ca; Tel.: +1-416-535-8501 (ext. 34201)
† These authors contributed equally to this work

Academic Editor: Firstname Lastname

Received: date
Revised: date
Accepted: date
Published: date

Citation: To be added by editorial staff during production.

Copyright: © 2025 by the authors. Submitted for possible open access publication under the terms and conditions of the Creative Commons Attribution (CC BY) license (https://creativecommons.org/licenses/by/4.0/).

Supplemental File S1

Supplemental File S1.1. RAMESES II reporting standards for realist evaluations: checklist

Table S1. RAMESES II reporting standards for realist evaluations: checklist

**TITLE**<br><br>Context, timing, and individualized care: A realist evaluation of safety planning for individuals living with suicide-related thoughts and behaviours, their families and friends and service providers			**Reported in document Y/N/Unclear/ Not applicable**	**Page(s) in document**
1		In the title, identify the document as a realist evaluation	Y	Page 1
2		Journal articles will usually require an abstract, while reports and other forms of publication will usually benefit from a short summary. The abstract or summary should include brief details on: the policy, programme or initiative under evaluation; programme setting; purpose of the evaluation;	Y	Page 1

**TITLE**<br><br>Context, timing, and individualized care: A realist evaluation of safety planning for individuals living with suicide-related thoughts and behaviours, their families and friends and service providers			**Reported in document Y/N/Unclear/ Not applicable**	**Page(s) in document**
		evaluation question(s) and/or objective(s); evaluation strategy; data collection, documentation and analysis methods; key findings and conclusions<br><br>Where journals require it and the nature of the study is appropriate, brief details of respondents to the evaluation and recruitment and sampling processes may also be included<br><br>Sufficient detail should be provided to identify that a realist approach was used and that realist programme theory was developed and/or refined		
3	Rationale for evaluation	Explain the purpose of the evaluation and the implications for its focus and design	Y	Pages 2-3
4	Programme theory	Describe the initial programme theory (or theories) that underpin the programme, policy or initiative	Y	Page 2
5	Evaluation questions, objectives and focus	State the evaluation question(s) and specify the objectives for the evaluation. Describe whether and how the programme theory was used to define the scope and focus of the evaluation	Y	Page 3-4
6	Ethical approval	State whether the realist evaluation required and has gained ethical approval from the relevant authorities, providing de-	Y	Page 4

**TITLE**<br><br>Context, timing, and individualized care: A realist evaluation of safety planning for individuals living with suicide-related thoughts and behaviours, their families and friends and service providers			**Reported in document Y/N/Unclear/ Not applicable**	**Page(s) in document**
		tails as appropriate. If ethical approval was deemed unnecessary, explain why		
7	Rationale for using realist evaluation	Explain why a realist evaluation approach was chosen and (if relevant) adapted	Y	Pages 3-4
8	Environment surrounding the evaluation	Describe the environment in which the evaluation took place	Y	Page 4-5
9	Describe the programme policy, initiative or product evaluated	Provide relevant details on the programme, policy or initiative evaluated	Y	Page 2, Page 5, Appendix A
10	Describe and justify the evaluation design	A description and justification of the evaluation design (i.e. the account of what was planned, done and why) should be included, at least in summary form or as an appendix, in the document which presents the main findings. If this is not done, the omission should be justified and a reference or link to the evaluation design given. It may also be useful to publish or make freely available (e.g. online on a website) any original evaluation design document or protocol, where they exist	Y	Pages 3-6
11	Data collection methods	Describe and justify the data collection methods – which ones were used, why and how they fed into developing, supporting, refuting or refining programme theory.<br><br>Provide details of the steps taken	Y	Page 5

**TITLE**<br><br>Context, timing, and individualized care: A realist evaluation of safety planning for individuals living with suicide-related thoughts and behaviours, their families and friends and service providers			**Reported in document Y/N/Unclear/ Not applicable**	**Page(s) in document**
		to enhance the trustworthiness of data collection and documentation		
12	Recruitment process and sampling strategy	Describe how respondents to the evaluation were recruited or engaged and how the sample contributed to the development, support, refutation or refinement of programme theory	Y	Page 4-5
13	Data analysis	Describe in detail how data were analysed. This section should include information on the constructs that were identified, the process of analysis, how the programme theory was further developed, supported, refuted and refined, and (where relevant) how analysis changed as the evaluation unfolded	Y	Pages 6-7
14	Details of participants	Report (if applicable) who took part in the evaluation, the details of the data they provided and how the data was used to develop, support, refute or refine programme theory	Y	Pages 7-9
15	Main findings	Present the key findings, linking them to contexts, mechanisms and outcome configurations. Show how they were used to further develop, test or refine the programme theory	Y	Pages 1119, Page 22, Appendix C
16	Summary of findings	Summarise the main findings with attention to the evaluation questions, purpose of the evaluation, programme theory and intended audience	Y	Pages 19-24; Appendix C

**TITLE**<br><br>Context, timing, and individualized care: A realist evaluation of safety planning for individuals living with suicide-related thoughts and behaviours, their families and friends and service providers			**Reported in document Y/N/Unclear/ Not applicable**	**Page(s) in document**
17	Strengths, limitations and future directions	Discuss both the strengths of the evaluation and its limitations. These should include (but need not be limited to): (1) consideration of all the steps in the evaluation processes; and (2) comment on the adequacy, trustworthiness and value of the explanatory insights which emerged<br><br>In many evaluations, there will be an expectation to provide guidance on future directions for the programme, policy or initiative, its implementation and/or design. The particular implications arising from the realist nature of the findings should be reflected in these discussions	Y	Page 25-26 and Appendix C
18	Comparison with existing literature	Where appropriate, compare and contrast the evaluation's findings with the existing literature on similar programmes, policies or initiatives	Y	Pages 19-22
19	Conclusion and recommendations	List the main conclusions that are justified by the analyses of the data. If appropriate, offer recommendations consistent with a realist approach	Y	Pages 22-24, Page 26, Appendix C
20	Funding and conflict of interest	State the funding source (if any) for the evaluation, the role played by the funder (if any) and any conflicts of interests of the evaluators	Y	Page 26

Adapted from table 1 in:

Wong, G.; Westhorp, G.; Manzano, A.; Greenhalgh, J.; Jagosh, J.; Greenhalgh, T. RAMESES II Reporting Standards for Realist Evaluations. BMC Medicine 2016, 14, 96, doi:10.1186/s12916-016-0643-1.

Supplemental File S2

Supplemental File S2.1. Consolidated criteria for reporting qualitative studies (COREQ): 32-item checklist

Table S2. Consolidated criteria for reporting qualitative studies (COREQ): 32-item checklist

No. Item	Guide questions/description	Reported on Page #
**Domain 1: Research team and reflexivity**		
Personal Characteristics		
1. Interviewer/facilitator	Which author/s conducted the interview or focus group?	Page 5
2. Credentials	What were the researcher's credentials? E.g. PhD, MD	Appendix B
3. Occupation	What was their occupation at the time of the study?	Appendix B
4. Gender	Was the researcher male or female?	Appendix B
5. Experience and training	What experience or training did the researcher have?	Appendix B
Relationship with participants		
6. Relationship established	Was a relationship established prior to study commencement?	Appendix B
7. Participant knowledge of the interviewer	What did the participants know about the researcher? e.g. personal goals, reasons for doing the research	Appendix B
8. Interviewer characteristics	What characteristics were reported about the interviewer/facilitator? e.g. Bias, assumptions, reasons and interests in the research topic	Appendix B
**Domain 2: study design**		
Theoretical framework		
9. Methodological orientation and Theory	What methodological orientation was stated to underpin the study? e.g. grounded theory, discourse analysis, ethnography, phenomenology, content analysis	Page 3-4, Appendix B
Participant selection		

10. Sampling	How were participants selected? e.g. purposive, convenience, consecutive, snowball	Page 4-5
11. Method of approach	How were participants approached? e.g. face-to-face, telephone, mail, email	Page 4-5
12. Sample size	How many participants were in the study?	Page 4-5, Page 7
13. Non-participation	How many people refused to participate or dropped out? Reasons?	Page 7
Setting		
14. Setting of data collection	Where was the data collected? e.g. home, clinic, workplace	Page 5-6
15. Presence of non-participants	Was anyone else present besides the participants and researchers?	Page 5
16. Description of sample	What are the important characteristics of the sample? e.g. demographic data, date	Pages 7-11
Data collection		
17. Interview guide	Were questions, prompts, guides provided by the authors? Was it pilot tested?	Page 5
18. Repeat interviews	Were repeat interviews carried out? If yes, how many?	Page 7
19. Audio/visual recording	Did the research use audio or visual recording to collect the data?	Page 5
20. Field notes	Were field notes made during and/or after the interview or focus group?	Page 5
21. Duration	What was the duration of the interviews or focus group?	Page 5
22. Data saturation	Was data saturation discussed?	Page 5
23. Transcripts returned	Were transcripts returned to participants for comment and/or correction?	Page 5
**Domain 3: analysis and findings**		
Data analysis		
24. Number of data coders	How many data coders coded the data?	Page 6
25. Description of the coding tree	Did authors provide a description of the coding tree?	Page 6

26. Derivation of themes	Were themes identified in advance or derived from the data?	Pages 6-7
27. Software	What software, if applicable, was used to manage the data?	Page 6
28. Participant checking	Did participants provide feedback on the findings?	N/A
Reporting		
29. Quotations presented	Were participant quotations presented to illustrate the themes/findings? Was each quotation identified? e.g. participant number	Pages 11-19
30. Data and findings consistent	Was there consistency between the data presented and the findings?	Pages 11-24
31. Clarity of major themes	Were major themes clearly presented in the findings?	Pages 11-24
32. Clarity of minor themes	Is there a description of diverse cases or discussion of minor themes?	Pages 11-24

Adapted from table 1 in:

Tong, A.; Sainsbury, P.; Craig, J. Consolidated Criteria for Reporting Qualitative Research (COREQ): A 32-Item Checklist for Interviews and Focus Groups. International Journal for Quality in Health Care 2007, 19, 349–357, doi:10.1093/intqhc/mzm042.
